# Assessment of knowledge and perceptions on leishmaniasis: An island-wide study in Sri Lanka

**DOI:** 10.1371/journal.pntd.0010821

**Published:** 2022-10-13

**Authors:** Rajika Dewasurendra, Hermali Silva, Nilakshi Samaranayake, Nuwani Manamperi, Nissanka de Silva, Panduka Karunanayake, Upul Senarath, Sanath Senanayake, Guofa Zhou, Nadira Karunaweera

**Affiliations:** 1 Department of Parasitology, Faculty of Medicine, University of Colombo, Colombo, Sri Lanka; 2 Department of Parasitology, Faculty of Medicine University of Kelaniya, Ragama, Sri Lanka; 3 Department of Zoology, Faculty of Applied Sciences, University of Sri Jayawardenapura, Gangodawila, Nugegoda, Sri Lanka; 4 Department of Clinical Medicine, Faculty of Medicine, University of Colombo, Colombo, Sri Lanka; 5 Department of Community Medicine, Faculty of Medicine, University of Colombo, Colombo, Sri Lanka; 6 University of California, Irvin, California, United States of America; Al-Jouf University College of Pharmacy, SAUDI ARABIA

## Abstract

Cutaneous leishmaniasis (CL) is a notifiable disease in Sri Lanka with increasing case numbers reported from every part of the country. In addition to disease treatment and vector control measures, knowledge and perceptions in a community are key contributors to a successful intervention program. An island-wide survey was carried out to assess the knowledge and perceptions regarding CL across the island, with 252 confirmed CL cases and 2,608 controls. Data was collected by trained personnel, using a pre-tested Case Reporting Form (CRF). Although the percentage who referred to CL by its correct name was low (1.4%), majority stated that it is a fly induced skin disease (79.1%). Knowledge on the symptoms, curability and the name of the vector was high in these communities, but specific knowledge on vector breeding places, biting times and preventive methods were poor. The patients were more knowledgeable when compared to the controls. Differences in the level of knowledge could be identified according to the level of education of the participants as well as across the different areas of the country. The main source of information was through the healthcare system, but the involvement of media in educating the communities on the disease was minimal. While this study population was unaccustomed to the use of repellants or sprays, the use of bed nets was high (77.7% of the participants) in this study population. Although misconceptions and incorrect practices are rare in Sri Lankan communities, promoting health education programs which may improve disease awareness and knowledge on vector and its control will further strengthen the control and prevention strategies.

## Author summary

Cutaneous leishmaniasis (CL) is a disease which causes skin ulcers and spread by the sand fly. In recent years, the number of CL patients reported from all parts Sri Lanka increased notably, indicating that the spread of the disease should be controlled. The general publics’ knowledge on the disease and its control methods, is very important to implement a successful control program. Thus, this study was conducted to assess the level of knowledge of the local community on CL, its vector and control methods. The required information was collected by interviewing CL patients as well as healthy persons. More than 50% of both groups gave correct answers to more general questions about the disease such as the disease name, symptoms and spread, indicating that the awareness of CL among the public is satisfactory. However, the study also indicated that the patients were more knowledgeable when compared to the healthy persons. Furthermore, it was evident that knowledge on more detailed information such as vector breeding places, vector biting times is poor in both groups and the healthcare system has been the primary source of information.

## Background

Leishmaniases are a group of parasitic diseases caused by *Leishmania spp*. These parasites are transmitted through the bites of infected female sand flies (*Phlebotomus spp*. and *Lutzomiya spp)*. There are 3 major forms of leishmaniasis namely cutaneous leishmaniasis (CL), mucocutaneous leishmaniasis (MCL) and visceral leishmaniasis (VL). CL is the most common form, that causes skin sores or skin lesions. MCL affects the mucosal tissues and VL,which affects the internal organs is considered the most serious form which has a case-fatality rate >95% in untreated cases [1]. VL, a neglected tropical disease, prevalent in Brazil, China, Ethiopia, India, Iraq causes 20,000–40, 000 deaths globally with 50,000–90,000 new cases, reported annually [2]. Majority of the MCL cases (>90%) occur in South America i.e., Bolivia, Brazil and Peru [3]. CL is prevalent in Afghanistan, Algeria, Bolivia, Brazil, Columbia, Iran, Iraq and Pakistan and it is estimated 60,000–1 million new cases occur worldwide annually [1,2].

In Sri Lanka, the predominant form is CL caused by *Leishmania donovani* zymodeme MON– 37, which is known to be responsible for VL in other parts of the world especially, in East Africa and India [4]. The first locally transmitted CL case was reported in 1992 [5] with a few isolated cases occurring over subsequent years until year 2000 [6]. However, the numbers reported have steadily increased during the past 2 decades making this a growing public health burden. Although most of the local leishmaniasis cases reported are CL, several VL and a few MCL cases has also been reported. [1]. CL is a notifiable disease (a disease that should be reported to the Epidemiology Unit, Ministry of Health, Sri Lanka; upon diagnosis) in Sri Lanka, with cases reported from all 25 administrative districts in the country [7].

In this backdrop, it is important to implement surveillance programs to enable effective control and elimination of this disease where diagnosis, treatment and vector control play a major role. However, delayed diagnosis, variable treatment seeking behavioral patterns, high exposure to sandfly bites in endemic areas have contributed to the increasing spread of disease [8]. The situation is compounded by the absence of a vaccine [9].

Although it is considered an established and an endemic disease, the knowledge regarding the disease and the vector is poor among the community, leading to a gap between the reported and actual annual numbers of cases [7], that will adversely affect disease surveillance programs and any efforts towards infection control. Little is known about the knowledge of general public on leishmaniasis and its vector. Studies assessing these are also scarce.

We studied the knowledge and perceptions on leishmaniasis and related aspects of the local communities residing in both high and low transmission areas across Sri Lanka.

## Methods

### Ethics statement

Ethical clearance for this study was obtained from The Ethics Review Committee, Faculty of Medicine, University of Colombo (EC– 17–062). Relevant approvals to conduct the health survey were received from the Director General of Health Services, Ministry of Health, Sri Lanka and from the respective Provincial Directors of Health Services and Regional Directors of Health Services.

The selected study participants were enrolled into the study upon obtaining written informed consent. Written proxy consent was obtained from the parent or the guardian in instances where the study subject is <18 years, together with the participant willingness to be included in the survey.

### Recruitment of study participants and data collection

Sample size of the index cases (n = 300) was calculated assuming 20% of controls having a risk factor / or exposure to leishmaniasis with an Odds Ratio (OR) of 1.5, using a two-tailed type 1 error of 5%, and a power of 80%. The number of controls were recruited using 1:5:5 case: neighbourhood: non-neighbourhood control ratio (i.e., 1500 neighbourhood controls residing within 500m radius of a patient and 1500 non-neighbourhood controls located beyond 1500m of patient’s residence).

The cases were selected randomly using sampling interval based on the national health administrative units. The country is divided into health administrative units that include Provincial health units (largest level), units at regional level (medium) and Medical officer of Health (MOH) level (smallest health administrative unit) that operates within the health care system. All the MOH areas (in all 9 provinces) in the island was initially included within the sampling frame. The MOH areas to be selected and the number of index cases to be included from each MOH area were calculated according to the case prevalence of each of the provinces as per patient incidence data recorded for 2018 [10]. After determining the number of index cases to be included in each MOH area, the patients to be included in the study were randomly selected from a list maintained at the MOH office, of leishmaniasis cases reported within the past 3 months of the scheduled visit. The patients selected were contacted with the aid of the Public Health Inspector (PHI) of each MOH. For each index case, 5 controls residing in the neighborhood (residing within<500 m) and 5 controls residing in a non-neighborhood area (residing >2KM) were randomly selected, with a total of 10 controls for each index case. Only the individuals over 14 years were selected for the study, as practiced previously [11,12] to enable reliable communication.

The residents of selected individuals were visited and a previously tested and validated Case Reporting Form (CRF), a written interviewer-administered questionnaire was completed by trained Research Assistants. The CRF was designed to capture data for a wider study with sections to gather data on risk factors, clinical features of the cases as well as to get information regarding participants’ knowledge and perceptions on the disease, the vector, and the prevention methods. The CRF was translated to Sinhala and Tamil languages which are the only 2 native languages used in Sri Lanka, by professional translators. The CRF was initially pre-evaluated by 6 individuals who were familiar with the disease, and pre-tested in a pilot study with 2 CL cases and 20 controls.

The hardcopy of the CRF was used to record the responses. Then the information was fed into the RedCAP (Research electronic data capture) software installed in Samsung Galaxy Tablets. This information was then uploaded into the central system on the same day, where they were electronically stored securely in a central database (S1 Table).

## Data analysis

Data analysis was done using SPSS (V 19.0). A score was assigned for each response in each question posed and a percentage score was calculated for the study population that reflects the level of knowledge and perceptions of the general public regarding the disease leishmaniasis. For the purpose of comparison of knowledge between the education groups, the study sample was divided into 4 education levels namely: level 1 = with no formal education or only primary education, level 2 = completed up to secondary education (Grade 6 to O/L), level 3 = with Advanced Level qualifications, level 4 = degree and above.

Chi squared test was used to test whether there are any significant differences between the sexes, occupation groups, education groups and the cases and controls in responses recorded against each question.

A knowledge-based score was developed for each study individual by assigning 1 point for each correct answer and 0 for each incorrect answer with a total score calculated for each individual (maximum score being 11, if the individual has answered all the questions correctly). According to the total knowledge score calculated for each individual, the study sample was divided into 3 categories viz. high, average and poor knowledge groups. The knowledge levels in each category were analyzed between cases/ controls, according to the education level, occupations, age categories and between the sexes using chi squared test. The mean and median knowledge scores between the aforementioned groups were tested using the t-test, ANOVA, Mann-Whitney U test and the Kruskall-Wallis test.

## Results

### General demographic characteristics of the selected study participants

The total study sample comprised 2860 individuals with 252 cases and 2608 controls that included 1278 neighborhood and 1330 non-neighborhood controls. The data from the 2 control groups were combined prior to the analysis. There were more females (n = 1787, 62.5%) than males (n = 1048, 36.6%) among study participants. Gender was not recorded in 25 (0.9%) individuals. Age range of the study sample was from 14 to 93 years (mean = 46.51 years; median = 46 years). Over 99% of the individuals belonged to the ethnic group *Sinhala*. Detailed socio–demographic characteristics of study participants is shown in Table 1.

**Table 1:** Socio-Demographic Characteristics of the study population.

Variable	Cases N (%)	Controls N (%)
**Gender**		
Male	146 (58.4)	902 (34.9)
Female	104 (41.6)	1683 (65.1)
**Age category**		
14–25	35 (13.9)	232 (8.9)
26–35	33 (13.1)	430 (16.5)
36–55	117 (46.4)	1124 (43.1)
56–70	58 (23%)	682 (26.2)
> 70	9 (3.6)	140 (5.4)
**Education category**		
No formal education/ primary education	7 (2.8)	45 (1.7)
Secondary education (up to O/L)	27 (10.8)	238 (9.2)
Grade 12 –A/L qualified	179 (71.3)	1792 (69.2)
Degree or above	38 (15.1)	514 (19.9)
**Occupation category**		
Unemployed	91 (36.1)	1589 (60.9)
Self-employed/ unpaid family worker	70 (27.8)	438 (16.7)
Farmer	18 (7.1)	122 (4.7)
Government employee	26 (10.3)	173 (6.6)
Private firm employee	19 (7.5)	81 (3.1)
Student	11 (4.4)	121 (4.6)
Retired	15 (6.0)	34 (1.3)

### Knowledge and perceptions on disease, disease transmission and the vector–differences between the cases and the controls

General knowledge on the disease, how the disease is transmitted and knowledge on the vector was assessed for the cases and the controls and is summarized in Table 2 (Table 2).

**Table 2:** Comparison of knowledge attributes between the cases and controls.

Knowledge variable	Response	Cases N (%)	Controls N (%)	Chi	p	OR
**Know the disease by the term**	Leishmaniasis	6 (2.4)	34 (1.3)			
	Fly induced skin disease	224 (90.3)	2037 (79.6)			
	Other	1 (0.4)	94 (3.7)			
	Not known	17 (6.9)	395 (15.4)			
Correctly identify the disease by name or as fly induced skin lesion		230 (92.7)	2071 (80.9)	21.43	>0.05	3.017
**Symptom of the disease**	Skin lesions	232 (92.1)	2083 (80.0)			
	Fever	2 (0.8)	39 (1.5)			
	Anemia	1 (0.4)	2 (0.1)			
	Enlarged liver and spleen	1 (0.4)	11 (0.4)			
	Not known	9 (3.6)	456 (17.5)	35.41	>0.05	-
**Complete cure possible**	Yes	224 (92.6)	1842 (75.0)			
	No	18 (7.4)	617 (25.1)	38.12	>0.05	4.168
**Leishmaniasis transmitted by**	Mosquito	0 (0.0)	17 (0.7)			
	Fly	8 (3.2)	95 (3.6)			
	Fruit fly	35 (13.9)	308 (11.8)			
	Sand-fly	174 (69.0)	1227 (47.1)			
	Direct contact	3 (1.2)	12(0.5)			
	Not known	29 (11.5)	928 (35.6)	65.76	>0.05	-
**Sandfly identification**	Yes	3 (1.2)	23 (0.9)			
	No	246 (98.8)	2530 (99.1)	0.204	0.654	1.32
**Know breeding places**	Yes	5 (2.0)	28 (1.1)			
	No	242 (98.0)	2522 (98.9)	1.576	0.209	1.83
					
**Know the biting times of sand-fly**	Daytime	1 (0.4)	5 (0.2)			
	Night	0 (0.0)	5 (0.2)			
	Dusk and dawn	9 (4.0)	32 (1.3)			
	Any time	0 (0.0)	4 (0.2)			
	Not known	213 (93.8)	2465 (98.2)	11.94	0.018	-
**Know vector control methods**	Mosquito nets	3 (1.2)	17 (0.7)			
	Insect repellants	1 (0.4)	9 (0.3)			
	Insecticide sprays	0 (0.0)	8 (0.3)			
	Meshing windows/ doors	0 (0.0)	3 (0.1)			
	Sanitation	5 (2.0)	16 (0.6)			
	Personal hygiene	2 (0.8)	14 (0.5)			
	Other	2 (0.8)	11 (0.4)			
	Not known	229 (90.9)	2449 (94.0)			
Identify the method/s of vector control correctly		4 (3.5)	37 (37.42)	0.053	0.816	-
**Information source**	Radio	0 (0.0)	2 (0.1)			
	Television	0 (0.0)	20 (0.8)			
	Print media	2 (0.8)	77 (3.0)			
	Friend/ neighbor/ co-worker	161 (63.9)	1887 (72.4)			
	Hospital/ healthcare worker	112 (44.4)	202 (7.8)	298.15	>0.05	-

Although the number of individuals who identified the disease by its correct name was low (cases = 6 (2.4%), controls = 34 (1.3%), whole study sample n = 40, 1.4%), majority of individuals of the study sample identified it as a fly-induced skin disease (cases = 224 (90.3%), controls = 2037 (79.6%), whole study sample n = 2261, 79.1%). A very high proportion (>80%) of the respondents named the symptoms of the disease as skin lesions (92.1% of the cases and 80% of controls) and over 75% of the respondents (92.6% of cases and 75% of controls) believed that the disease is curable. Though nearly half of the study sample correctly stated that leishmaniasis is transmitted by the sand fly (cases = 69%, controls = 47.1%, whole study sample n = 1379, 48.2%), over 95% of the individuals (both cases and controls) stated that they cannot identify a sand fly correctly and the knowledge on vector breeding places, biting times and vector control measures were very poor (Table 2).

Statistical analyses were carried out to determine whether there is a significant difference between knowledge regarding leishmaniasis between cases and controls (Table 2).

The percentage of study participants that correctly identified the disease by its name (or as a fly induced skin disease), the symptoms of the disease, believed the disease is completely curable, and knew the name of the vector correctly as the sand fly were significantly higher among the cases when compared to the controls (Chi squared test, p<0.05) (Table 2).

However, the percentages of cases and controls with a knowledge on vector breeding places, biting times and vector control measures were very low and were not significantly different between the cases and controls (Table 2).

The main source of information for the controls were their friends, co-workers or the neighbours, while more than half of the cases (54.1%) reported that they got the information on the disease through the health care system. None of the cases reported that the knowledge of the disease was gained through electronic or print media, and very few of the controls (n = 35, 1.9%) said that their source of information was either radio, television or printed media.

Based on the total knowledge score of each individual they were grouped into 3 groups namely; individuals with high, average and poor levels of knowledge. The percentages with high level of knowledge were very low in both cases (0.1%) and controls (0%), but the percentages with average level of knowledge were significantly high in cases (61.1%) when compared to that of controls (37.7%) (Chi = 52.55, df = 2, p<0.05).

The mean knowledge score of the cases (mean = 3.492) was significantly high when compared to the controls (mean = 2.844) (t = -7.448, df = 2806, p<0.05, 95% CI = ±2.367).

## The differences of knowledge based on level of education

The differences in responses between the individuals with different education levels were tested for these knowledge components.

**Fig 1:**
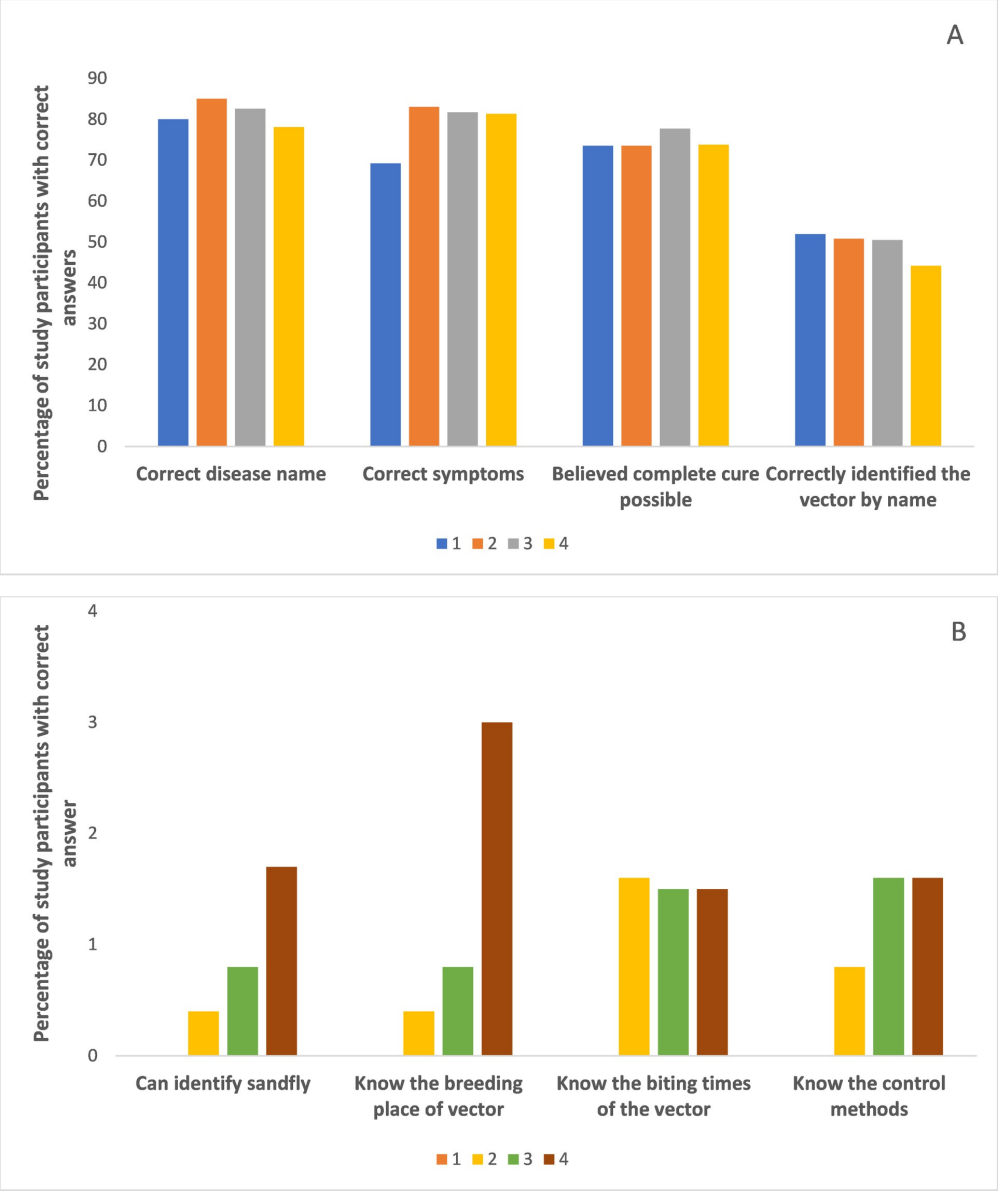
Differences in knowledge according to the education group. The study participants were categorized into four education groups. Fig 1A – Knowledge on disease related facts/Fig 1B – knowledge on vector related facts. Four education levels were compared. 1= No formal education or only primary education: 2= Secondary education: 3=Up to Advanced level education: 4=Degree or above.

Of the individuals who could identify the disease name correctly or indicated that it is a fly induced skin disease (n = 2298), 70.3% were with Advanced Level qualifications (level 3). This was significantly higher (Chi = 10.78, df = 3, p = 0.013) than the percentage of individuals with level 1 education (1.7%), level 2 education (9.6%) or the level 4 education (18.3%) (Fig 1A).

The percentage of individuals with A/L qualifications (level 3) who named the vector correctly as the sand fly was significantly higher (71.3%) when compared to those in other education levels i.e., 1.8% in level 1, 9.6% in level 2 and 17.3% in level 4 (Chi = 30.36, df = 15, p = 0.011).

A similar percentage of individuals (48.5%) with level 3 education and level 4 education correctly identified the breeding places of sandflies. These percentages were significantly high when compared to the percentages of individuals with level 1 or 2 education levels (Chi = 18.907, df = 3, p<0.05).

However, percentages of individuals with the ability to identify the sand fly, knowledge on biting time, control methods of sand fly and symptoms and the percentages of individuals who believed that the disease could be cured completely were not significantly different across the education groups. Similarly, the source of information also was comparable between the different education groups (Fig 1B).

## The differences of knowledge between the administrative units

**Fig 2:**
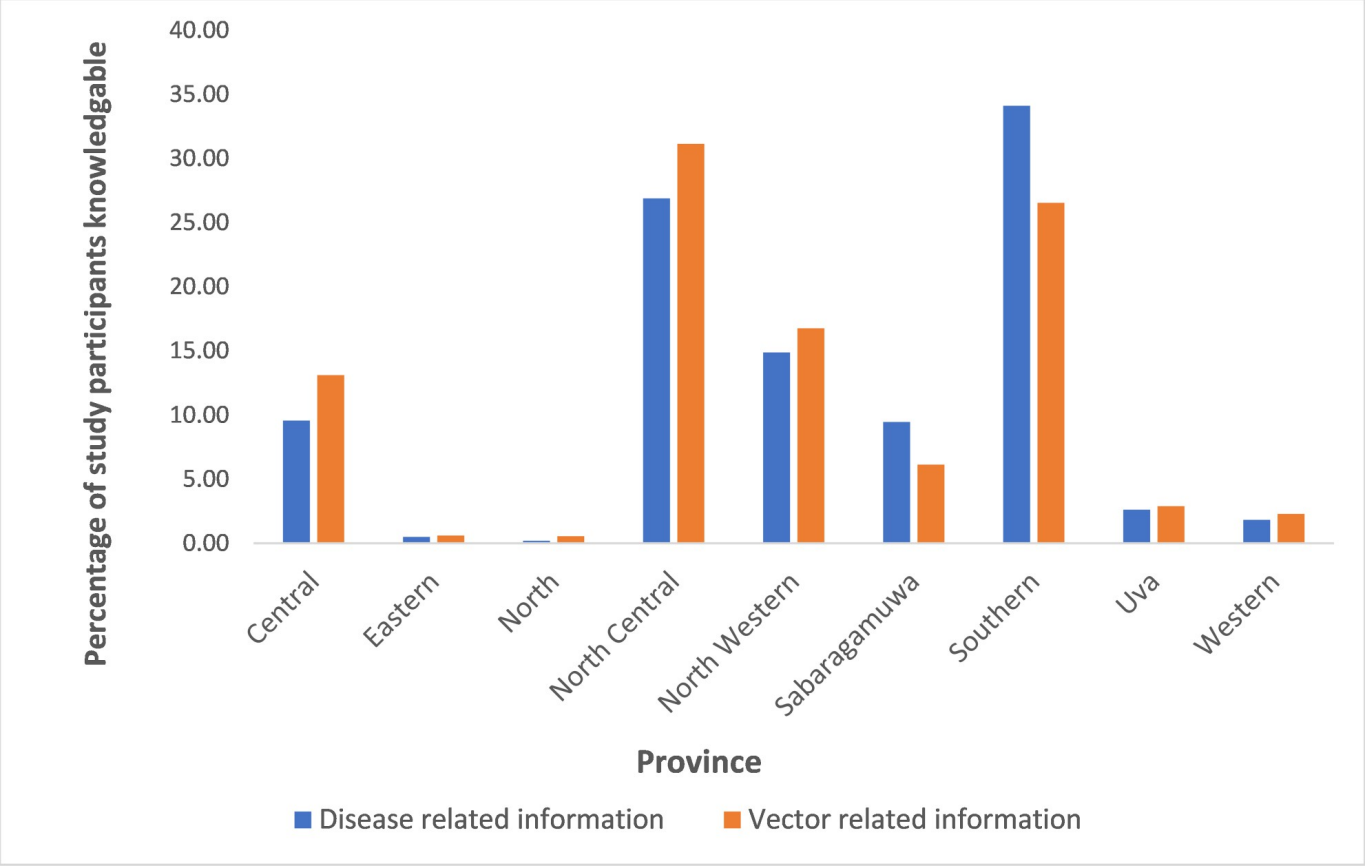
Differences in knowledge according to the provinces.

The highest mean knowledge scores for MOHs, districts and provinces were from Rambukkana MOH area (mean score = 4.00), Kegalle district (mean score = 4.00) and the Eastern province (mean score = 3.72) while the lowest mean knowledge scores were for Gampaha MOH (mean score 0.54), Vavuniya district (mean score = 1.66) and for the Northern Province (mean score = 1.66) (Fig 2). The correlation between the mean scores and the incidence rates of each district and MOH areas were assessed. The correlation between the mean knowledge scores and the number of cases reported either from the selected MOH areas in 2018 (r^2^ = 0.108, p = 0.252) or from the districts (r^2^ = 0.001, p = 0.805) were not significant.

However, the mean knowledge scores between the districts differed markedly (t = 3.26, df = 442, p = 0.001) and so did the values between provinces (t = 2.4, df = 11, p = 0.035).

The responses of males and females on areas of knowledge tested in this study were comparable (p>0.05). Similar pattern was seen between the occupation groups and the age groups (p >0.05). Furthermore, there were no significant differences of score levels (high/ medium/ low) or the mean knowledge score between the sexes (t- test), age groups, education groups or the occupation groups (ANOVA).

## Behavioral determinants of risk of acquiring leishmaniasis among the study participants

The behaviors and practices among the study sample, which may lead to increased risk of acquiring the disease or protection from insect bites, hence the disease were also studied.

Over 98% of the respondents stated that they slept indoors and the majority (96.1%) slept on a bed. The use of mosquito nets was prominent among these communities (77.7%). The practice of visiting jungle areas for firewood, ‘*chena*’ cultivation or visiting water bodies were not regular practices for the individuals in this study sample with only 10.2% mentioning that they visit the jungle and only 14.7% mentioning that they visit the water bodies.

Although the above-mentioned factors reduce the risk of acquiring the disease, there were some practices among these study participants, which could increase the risk as well.

Most individuals (87.4%) have travelled to high disease incidence areas (annual case numbers >600; [7] within the past 5 years. The use of insect repellents or spraying of insecticides inside/ outside the house was uncommon. Only 28.7% said that they use insect repellants and a lesser percentage (1.7%) stated that they spray insecticides either inside or outside the housing unit that they occupy. Both females and males stated that they rarely use attires that cover their whole body when working outside.

None of the leishmaniasis patients in the study sample have sought treatment from traditional healers.

## Discussion

Leishmaniasis is a disease that was established within the past 2 decades in Sri Lanka, and has progressed steadily, spreading across the country, creating a significant health threat [1]. Emergence of virulent clinical forms e.g., VL and MCL add to the burden making preventive and control measures a priority. Sri Lanka developed a National Action Plan for leishmaniasis control in 2010, which was updated in 2014, which sets the stage for implementation of control measures against leishmaniasis. The role of active participation of community members would be important for a successful control programs as highlighted previously and has gained attention [13]. Furthermore, understanding the levels of education, social categories as well as the attitudes and beliefs in a community, helps the effective implementation of a successful control program. Lack of awareness of the disease, how it is transmitted, and details on preventive measures could weaken the preventive efforts. Furthermore, ignorance regarding the role of sand flies in transmitting the disease, sand fly biting time, role of insect repellants, sand fly bite prevention and using personal protection gears (e.g., attire suitable for outdoor activities) as well as lack of awareness regarding medical treatment may have negative effects upon control efforts [14].

Thus, studies to evaluate the status of knowledge of the disease and that of the vector, attitudes towards the control methods, preventive measures and treatment as well as practices that may increase the disease burden (or reduce the risk) are of relevance and importance in gaining information to set the stage for implementation of control measures. This study was conducted as a nation–wide survey covering 74 MOH areas, in 14 administrative districts, in all 9 provinces to assess the knowledge, attitude and practices of both patients who were infected with leishmaniasis as well as of apparently healthy individuals who lived either in the neighbourhood or at a physical distance beyond 2 Km from known patients. This study was conducted as a part of a large case–control study designed to evaluate the risk factors, host genetic factors and serology. Therefore, a common CRF, which included the components to identify knowledge and perceptions of the study participants, was used to record the responses of the participants.

Though the overall percentage of individuals who identified the disease by its exact name was very low (1.4%), it is encouraging to note that most of the study participants identified the disease as one introduced following an insect bite. Moreover, a very high proportion of study participants knew the symptoms of the disease correctly and believed the disease is curable indicating a satisfactory level of basic knowledge of the disease in the community, compared to the knowledge level of many other countries where knowledge on origin of the disease, transmission method etc. are very poor [15–18].

Nearly half of the study participants (48.2%) could identify the vector by its local name “*weli massa*” or the sand fly. This is impressive when compared to the knowledge on the disease transmission of other CL affected communities in the South Asian region and elsewhere with only 9.2% of the study participants in Punjab, Pakistan being able to identify the sand fly as the vector of leishmaniasis [19], while in Sothern Iran [20] and Kerala, India [21] none of the respondents could name the vector correctly. Furthermore, in most instances communities from other parts of the world assumed an association between leishmaniasis and mosquito bites [20].

Although the basic knowledge of the disease and the vector seems to be at a higher level in Sri Lanka, ability to identify the vector, knowledge regarding its breeding places, biting times and vector controlling methods were poor. This indicates that information related to the vector aspects and its control have failed to reach these communities exposing a void in the information flow. Identification of sand flies by the naked eye in any case is difficult. In a KAP study done in Isfahan, Iran involving students, almost 98% of the participants were aware of the involvement of sand flies in infection transmission, but only 28.6% of the participants were able to identify the sand fly [22].Thus the low percentage (0.9%) of the study participants who could identify the vector is not surprising. Increasing the awareness on vector breeding places, biting times and vector control methods are important to ensure community support in a future national effort towards disease prevention and control.

A significantly higher percentage of individuals who is having/ or have acquired the disease previously (cases) made correct responses for 5 out of 7 knowledge components tested compared to those who did not have the disease currently or previously (controls). Similarly, the mean knowledge score of the cases were significantly higher when compared to the controls. Moreover, the proportion of cases with an average knowledge on leishmaniasis was significantly higher than among the controls. All these indicate that the knowledge acquisition of the cases in this study group has happened through the health care systems. This argument can be further confirmed by the fact that the knowledge components tested, the total knowledge score and the knowledge level (low/medium/high) were not significantly different between the age groups, gender groups or between the occupation groups. Similarly, nor were they significantly different between the education groups except for few knowledge components (i.e., identification of the disease, identification of the vector and its breeding place), indicating the basic knowledge the community has acquired is unlikely to have happened through standard education system or reading educational material, but by having a leishmaniasis patient in the family or the neighbourhood. Furthermore, very few of the participants stated that the source of knowledge was printed or electronic media, which highlight the barely used or untapped resources for transfer of information or healthcare messages on leishmaniasis. Additionally, it is interesting to note, that there was no significant difference on the knowledge scores or the knowledge level between the neigbhourhood and non-neighbourhood controls when analyzed separately, indicating that although there is a CL patient in the community, the knowledge gained by the patient and the family has not transmitted effectively to the community.

However, there was a significant relationship between the education level and identifying the disease and the vector correctly with such ability increasing with the level of education up to the Advanced level qualifications. Surprisingly, a lesser proportion of individuals with basic degree qualifications (or higher) could identify the disease or the vector. The main reason for this could be most participants of this survey may have acquired degrees in the fields of Arts or Commerce with zero inputs in biological sciences. This is in spite of the fact that there were a comparatively higher proportion of individuals who have followed Biological Sciences up to Advanced Level examination, but failed to continue their higher studies in that field, which highlights the lack of relevant knowledge retained from school education system with the shift in focus at later stage.

Another interesting feature that was seen in the study sample is the tendency of accepting that they do not know the right answer, without guessing an answer on such occasions, though they had that choice. This is evident from the low percentages of individuals who gave incorrect answers to the questions (most of the individuals either gave the correct answer or acknowledged that they do not know the answer). This will be a plus point when conveying the correct information to these communities as most of the individuals will not bear misinformation or beliefs regarding the disease or the vector, which are often difficult to change when such views are embedded in communities [18].

Alidosti *et al*., in 2021 reported that an individual’s correct understanding of seriousness, risks as well as awareness on origin, transmission, prevention and correct treatment of the disease will affect on how a person will respond to the disease which could result in people paying more attention to preventive behaviours [14]. On the other hand negative perceptions and misconceptions will cause people to take inappropriate actions [16].

Unlike in other geographical regions, use of mosquito nets is a common practice in most areas of Sri Lanka, which could be related to the high burden of mosquito borne diseases, especially malaria and dengue. Furthermore, long lasting insecticide impregnated bed nets (LLINs) was a strategy used in the malaria elimination campaign in Sri Lanka. Therefore, the high usage of mosquito nets with or without insecticide impregnation, could be considered as a common practice, to gain protection over many of the mosquito/ insect-borne diseases, particularly effective, if the vector predominantly bites indoors.

Similarly, indoor residual spraying of insecticides was a practice when malaria control and elimination programs were underway in the past. Such intensive programs prior to malaria elimination in year 2016 may have curtailed sand fly population also, as a collateral benefit [1]. Though the practice of indoor residual spraying ceased with the declaration of elimination of malaria in Sri Lanka, the regular but limited-scale use of insecticides by residents either as sprays or smoke might have a similar but less potent effect.

Although the information regarding treatment-seeking behavior is available for leishmaniasis patients in the study group, the attitude and beliefs of the controls towards treatment of leishmaniasis could not be assessed, which may be viewed as a shortcoming of this survey. However, it is of interest to note that none of the index cases sought traditional medicine or attempt self-medication before coming to a medical clinic. This finding contrasts with those from other parts of the world where the communities either use traditional methods and/ or self-medication instead of seeking standard western medicine. Ramdas *et al*. in 2012 reported the proportion of CL patients who self-medicated the lesions with harmful substances before diagnosis was as high as 76% [15].

The majority of the study participants in the control group were females (>65%). This could be due to the higher participation of females in the survey due to their availability at their residences while the majority of males may have departed for their workplaces. The recent labour force survey annual report data (2019), which indicates the male: female percentage ratio in active labour force as 73: 34.5 strengthens this assumption [23], which however, may be viewed as a limitation. Furthermore, the fact that majority of the survey participants being housewives could also be the reason for the higher unemployment rate of this study sample (58.7%).

## Conclusions

This study shows the majority of patients and apparently healthy controls had reasonable levels of knowledge on the disease and the vector, but the knowledge on general topics i.e., name of the disease, vector and symptoms were significantly high in cases compared to the controls. It is also evident that more specific and more detailed information (e.g., breeding places and biting time of the vector) have not reached the communities (either cases or controls). Involvement of print and electronic media in conveying information regarding the disease is minimal. Education through television programs (interviews, documentaries), social media and print media remain as untapped sources for effective communication regarding leishmaniasis, particularly those related to vector breeding sites and preventive measures that will facilitate future control programs.

## Supporting information

S1 Table Data base file. All the data used for the analyses in this study is available through this.
(XLSX)
